# Alterations in Nitric Oxide Production After Post-Weaning Social Isolation

**DOI:** 10.33549/physiolres.935723

**Published:** 2025-12-01

**Authors:** Stanislava VRANKOVA, Zuzana GALANDAKOVA, Jana KLIMENTOVA, Olga PECHANOVA, Martina CEBOVA

**Affiliations:** 1 Centre of Experimental Medicine Slovak Academy of Sciences, Institute of Normal and Pathological Physiology, Bratislava, Slovak Republic; 2 Institute of Pathophysiology, Faculty of Medicine, Comenius University, Bratislava, Slovak Republic

**Keywords:** Social isolation, Nitric oxide, Oxidative stress

## Abstract

Early-life stressful stimuli, such as social isolation, alter brain neurochemistry and lead to negative behavioral outcomes in adulthood. Isolated animals are deprived of social interactions, which results in impaired brain development. Post-weaning isolation rearing deregulates various brain processes and may affect nitric oxide (NO) signaling. The aim of our study was to determine time-dependent impact of social isolation on behavioral and biochemical parameters in Wistar Kyoto rats. At the age of 21 days, male rats were randomly assigned into four groups reared in isolation or socially for 10 or 29 weeks. At the end of the rearing, open-field and prepulse inhibition (PPI) tests were carried out. Furthermore, in several brain areas we assessed NO synthase (NOS) activity, protein expression of nNOS and iNOS isoforms and the concentration of conjugated dienes (CD), a marker of lipid peroxidation. The number of entries into the central zone of the open field test decreased significantly only after 29 weeks of isolation. Isolated rats (IR) rats exhibited impaired habituation of the acoustic startle response after prolonged social isolation. While cerebellar NOS activity and nNOS protein expression decreased significantly in IR rats after 29 weeks of isolation, the expression of nNOS and iNOS was increased in the hippocampus. 10-week and 29-week social isolation led to increased CD concentration in the brain. Our results suggest that the duration of social isolation plays an important role in the development of behavioral and biochemical changes in the brain. The decreased NO bioavailability may result from lipid peroxidation, oxidative stress, and inflammatory responses.

## Introduction

Several studies have shown that disruptions in brain development caused by stressful experiences in the early stages of life may trigger serious psychiatric disorders in adulthood. Rats reared in social isolation exhibit various cognitive, behavioral, and neurochemical abnormalities resembling to several core symptoms of schizophrenia [1,2]. For social animals, post-weaning social isolation is a strong stressor due to the deprivation of critical neurobiological development stimuli.

Prepulse inhibition (PPI) is commonly used as an operational measure of a process called “sensorimotor gating”. Gating refers to a basic cognitive process, by which excess or trivial stimuli are screened or “gated out” of awareness. Due to inefficient gating aberrant salience can be attributed to irrelevant stimuli, which is considered as a core psychopathological mechanism of psychosis [3]. The PPI of the startle response is an important measure of information processing deficits and inhibitory failure in patients with schizophrenia. PPI occurs in the same lawful manner in all mammals, from humans to rodents, making it an ideal candidate for cross-species translational research [4].

The literature shows that schizophrenic patients have elevated prooxidant substances, reduced antioxidant capacity, and high levels of proapoptotic markers [5,6]. Impaired antioxidant defense systems and increased lipid peroxidation have been reported in peripheral tissues and brain samples of patients with schizophrenia. An increased concentration of malondialdehyde has been observed in the plasma and red blood cells [7,8]. Additionally, studies using magnetic resonance spectroscopy have shown that GSH levels were reduced by ~50 % in the prefrontal cortex of drug-naïve patients with schizophrenia [9]. Variability in glutamate cysteine ligase (GCL), the rate-limiting enzyme of GSH synthesis was found to be associated with schizophrenia [10].

Nitric oxide is an important signalling molecule in the nervous system. NO is involved in neuronal migration during brain development, the formation of synapses, and the regulation of neurotransmission by influencing neurotransmitter release [11,12]. Alterations in NO production and/or nNOS expression have been associated with the etiopathology of various psychiatric diseases, including schizophrenia [13]. Moreover, significant disturbances in NO levels in the brain structures were revealed in patients with schizophrenia [14].

The role of the cerebellum in schizophrenia has been highlighted by recent studies, which suggested an impaired sequencing and coordination of sensorimotor and mental processes [15,16]. The cerebellum is connected to many areas of the cerebral cortex via cortico-cerebellar-thalamic-cortical circuit. Disturbances in these circuits may be implicated in cognition. Reduced cerebellar volume, decreased blood flow and dysfunctional cortical pathway were observed in schizophrenic patients [17,18]. There is also increasing evidence supporting that abnormalities in the hippocampus, which subserve a range of roles in learning, memory, and emotional regulation, are associated with the symptoms and cognitive impairment of schizophrenia [19].

In this study, we investigated whether 10- or 29-week social isolation rearing is related to behavioral changes and changes in the redox-oxidative balance or nitric oxide signaling in the cerebellum and the hippocampus of Wistar Kyoto rats.

## Material and Methods

### Animals

All experimental procedures were performed in accordance with the guidelines of the Institute of Normal and Pathological Physiology, Centre Experimental Medicine Slovak Academy of Sciences (INPP CEM SAS), and were approved by the State Veterinary and Food Administration of the Slovak Republic (Ro-591/17-221) and by an ethics committee according to the European Convention for the Protection of Vertebrate Animals used for Experimental and Other Scientific Purposes, Directive 2010/63/EU of the European Parliament. Male Wistar Kyoto rats (WKY) 13- week-old and 32-week-old, were used for the investigation (n=8 in each group).

Adult timed-pregnant Wistar Kyoto rats (Velaz, Prague, Czech Republic) arrived at the animal facility on gestational day 16. Approximately a week later, the litter was born. Rats were kept under standard housing conditions with a constant 12:12 h light/dark cycle, temperature (22 ± 2 °C) and humidity (55 ± 10 %). Food and water were available *ad libitum*. At the end of these periods, the behavioral tests were carried out, the animals were sacrificed and the brain was isolated and processed for biochemical analyses.

### Social isolation

The sample involved 32 male rats. The animals were separated from their mothers after weaning (21 days postnatal) and were randomly divided into four groups. Two experimental groups were subjected to either 10-week or 29-week isolation (single rat per cage, 43.5 x 28 x 23 cm); in two control groups, rats were reared socially (SR) for 10 or 29 weeks (three rats per cage, 55.5 x 34.5 x 19.5 cm). In each group, the rats were able to see, smell and hear other animals in the room.

### Behavioral testing

The behavioral tests included measurements of exploratory behavior in open field test, PPI and habituation of the startle reflex. We used a standard protocol for assessing locomotor behavior of rats in the open field [20]. Measurements were carried out using a video-based system AnyMaze. Gating was measured using the prepulse inhibition of the acoustic startle response (PPI). In PPI, a weak acoustic stimulus presented shortly before a strong startling sound attenuates the startle response. We used a standard protocol of PPI assessment in rats [21] and a startle box (Med Associates, UK) with calibrated loudspeakers and accelerometric measurement of motor response. Stimuli (white noise) had the intensity of 3, 6, 12 dB (prepulse) a 55 dB (pulse) above the level of continuously presented background noise (white noise, 65 dB). Duration of prepulse was 20 ms and pulse 40 ms. Prepulse-to-pulse interval was 100 ms. In a randomized sequence 10 "pulse-alone" stimuli and 5 prepulse-pulse stimuli of each prepulse intensity were presented. Mean interstimulus interval was 15 s. PPI was calculated as: PPI = (1-PP/PA)*100 %, where PA is mean amplitude in the pulse-alone trials and PP is mean amplitude in the prepulse-pulse trials.

### Conjugated dienes concentration

The concentration of conjugated dienes (CD) was measured in lipid extracts of the frontal cortex. Samples were homogenized in 15 mmol/dm3 EDTA containing 4 % NaCl. Lipids were extracted using a 1:1 chloroform-methanol mixture. Chloroform was evaporated in the N_2_ atmosphere and after the addition of cyclohexane, conjugated diene concentrations were determined spectrophotometrically (λ = 233 nm, NanoDropTM 2000c, UV-Vis spectrophotometer, Thermo Fisher, Waltham, MA, USA). The concentration of CD was expressed as nmol per g tissue.

### Cytokines concentration

Cytokine levels were determined by Bio-Plex Pro™ (Bio-Rad, Hercules, CA, USA) rat cytokine, chemokine, and growth factor assays in plasma.

### Total activity of NO-synthase

Total NO synthase activity was determined in crude homogenates of the cerebellum and hippocampus by measuring [^3^H]-L-citrulline formation from [^3^H]-L-arginine (ARC, St. Louis, MT, USA) as described elsewhere [22,23]. [^3^H]-L-citrulline was measured with the Quanta Smart triCarb Liquid Scintillation Analyzer (Packard Instrument Company, Meriden, CT, USA). NOS activity was expressed as pkat/min per gram of protein.

### Western blot analysis

For Western blot analysis, samples of the tissues (cerebellum and hippocampus) were homogenized in a lysis buffer, 0.05 mM Tris containing protease inhibitor cocktail (Sigma-Aldrich, Taufkirchen, Germany). Protein concentrations were determined by Lowry assay. Proteins were subjected to 10 % SDS-PAGE and transferred onto a nitrocellulose membrane. Membranes were blocked with 5 % non-fat milk in Tris-buffer solution (TBS; pH 7.6) containing 0.1 % Tween-20 (TBS-T) for 1 h at room temperature and then incubated in the presence of the appropriate primary antibodies overnight at 4 °C with polyclonal rabbit anti-neuronal NOS, anti-inducible NOS and anti-GAPDH (as control) antibodies (Abcam, Cambridge, UK). Antibodies were detected using a secondary peroxidase-conjugated antirabbit antibody (Abcam, Cambridge, UK). The bands were visualized using the enhanced chemiluminescence ECL system (Bio-Rad, Hercules, CA, USA), quantified by using ChemiDoc™ Touch Imagine System (Image Lab™ Touch software, Bio-Rad, Hercules, CA, USA), and normalized to GAPDH bands.

### Statistical analysis

Data were processed and analyzed using Statistica. All data were analyzed by two-way analysis of variance (ANOVA) with factors rearing condition (social vs. isolation) and rearing duration (10 week vs. 29 week) followed by Bonferroni post-hoc test when appropriate. The level of statistical significance was set as p < 0.05.

## Results

### Biometrical parameters

Body weight did not change after 10 weeks of isolation (SR:316.55 ± 5.8 g vs. IR:311.77 ± 5.2 g). However, after prolonged isolation, a significant increase in body weight was observed in IR rats compared to SR rats (513.14 ± 5.5 g vs. 547.43 ± 8.2 g; p < 0.05).

### Open field test

**Fig. 1:**
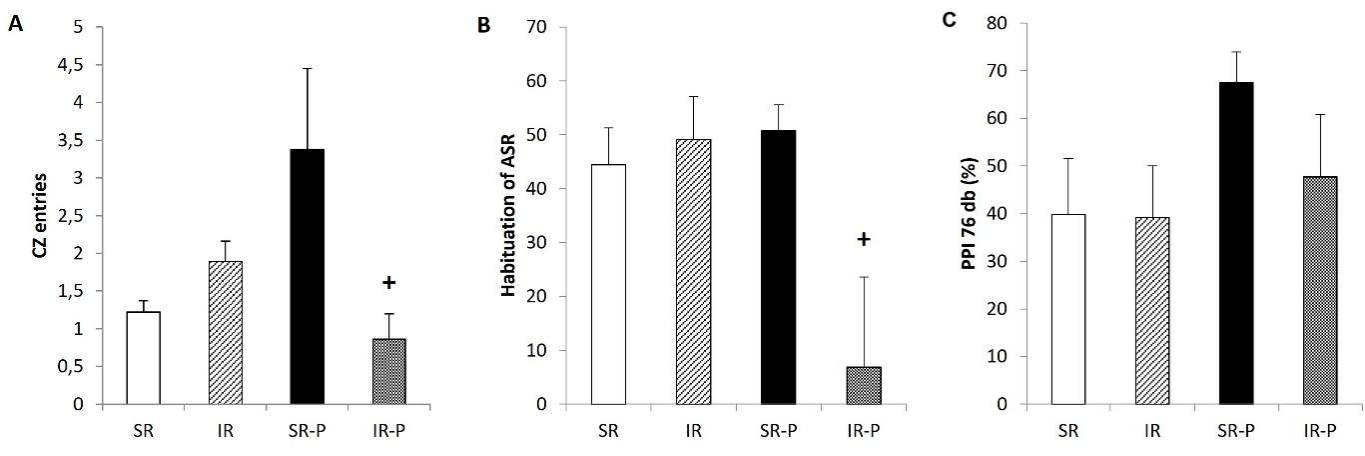
Central zone entries in the EPM test (**A**), habituation of acoustic startle response (**B**) and prepulse inhibition of the acoustic startle response (**C**) at the prepulse intensity 76 db. Results are expressed as mean ± SEM (+ p < 0.05 vs. SR-P). SR: socially reared rats; IR: isolated reared rats; SR-P: socially reared rats – prolonged rearing; IR-P: isolated reared rats – prolonged isolation; CZ, central zone; ASR, acoustic startle response; PPI, prepulse inhibition.

The number of entries into the central zone (CZ) in the open field test did not change significantly after 10 weeks of social isolation. However, prolonged isolation revealed a significant decrease in entries into the CZ (p < 0.05) in the group that was reared in social isolation (Fig. 1A). This indicates that the rats were less active during the test and spent less time in the central area.

### Startle habituation and prepulse inhibition of startle (PPI) paradigm

Startle habituation was not changed after 10 weeks of isolation rearing, but after 29 week of isolation it was significantly decreased in IR rats (p < 0.05) compared with SR rats (Fig. 1B). Rearing conditions or duration of social isolation had no significant effect on the prepulse inhibition (Fig. 1C).

### Cytokines concentration

**Fig. 2:**
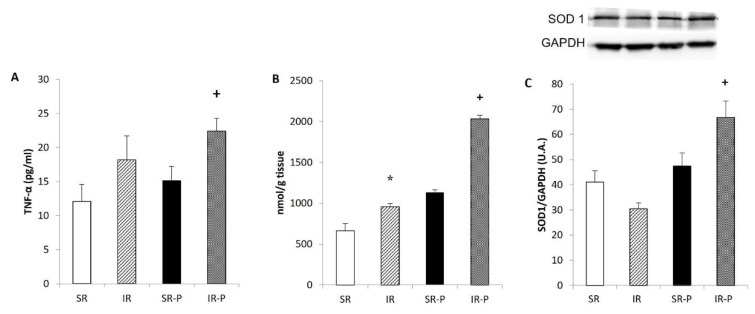
Concentration of TNFα in plasma (**A**), concentration of conjugated dienes in the brain cortex (**B**) and protein expression of SOD 1 (**C**) in the hippocampus. Results are expressed as mean ± SEM (* p < 0.05 vs. SR; ^+^ p < 0.05 vs. SR-P). SR: socially reared rats; IR: isolated reared rats; SR-P: socially reared rats – prolonged rearing; IR-P: isolated reared rats – prolonged isolation; SOD1, Superoxide dismutase 1; GAPDH, glyceraldehyde 3–phosphate dehydrogenase; A.U., arbitrary units.

The concentration of TNF-α did not change after 10 weeks of social isolation. However, TNF-α levels were significantly higher in isolated compared to socially reared animals after 29 weeks of isolation (p < 0.05) (Fig. 2A). The duration of rearing did not affect other cytokines, chemokines, or growth factors (data not shown).

### Conjugated dienes concentration

As shown in Fig. 2B, concentration of CDs in the frontal cortex was significantly affected by social isolation and duration of rearing (p < 0.05), showing significantly higher levels in animals reared for 29 weeks.

### Total activity of NO-synthase

**Fig. 3:**
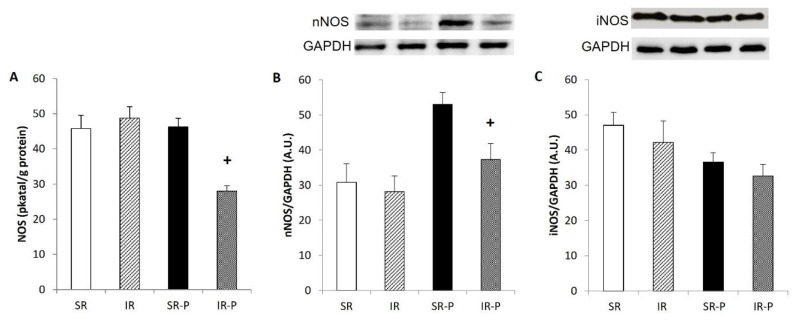
Total NOS activity (**A**) protein expression of neuronal NOS (**B**) and inducible NOS (**C**) in the cerebellum. Results are expressed as mean ± SEM (**^+^** p < 0.05 vs. SR-P). SR: socially reared rats; IR: isolated reared rats; SR-P: socially reared rats – prolonged rearing; IR-P: isolated reared rats – prolonged isolation; nNOS, neuronal nitric oxide synthase; iNOS, inducible nitric oxide synthase; GAPDH, glyceraldehyde 3–phosphate dehydrogenase; A.U., arbitrary units.

**Fig. 4:**
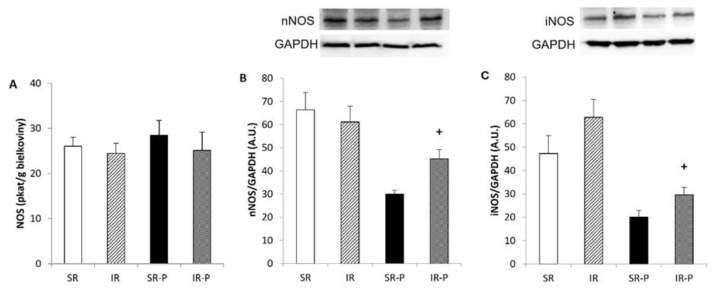
Total NOS activity (**A**) protein expression of neuronal NOS (**B**) and inducible NOS (**C**) in the hippocampus. Results are expressed as mean ± SEM (**^+^** p < 0.05 vs. SR-P). SR: socially reared rats; IR: isolated reared rats; SR-P: socially reared rats – prolonged rearing; IR-P: isolated reared rats – prolonged isolation; nNOS, neuronal nitric oxide synthase; iNOS, inducible nitric oxide synthase; GAPDH, glyceraldehyde 3–phosphate dehydrogenase; A.U., arbitrary units.

The total NOS activity in the cerebellum was affected by the duration of rearing (p < 0.05), being significantly lower after 29 weeks of rearing (Fig. 3A). There was no significant effect on NOS activity in the hippocampus (Fig. 4A).

### Western blot analysis

The protein expression of superoxide dismutase 1 (SOD1) was not changed after 10 weeks of isolation. (Fig. 2C). When compared with socially housed animals, isolated rats showed significantly increased SOD 1 protein expression in the hippocampus after 29 weeks of rearing (p < 0.05). Rearing duration had a significant effect on nNOS expression in the cerebellum (Fig. 3B) and the hippocampus (Fig. 4B), but the effects were in the opposite direction. In the cerebellum, nNOS expression was significantly decreased after 29 weeks of social isolation compared with socially housed animals (p < 0.05). In the hippocampus, in contrast, 29 weeks of rearing resulted in increased nNOS expression when compared with socially-reared animals (p < 0.05).

Protein expression of iNOS in the cerebellum was not significantly influenced by rearing conditions or duration of rearing (Fig. 3C) in animals reared in social isolation. As shown in Fig. 4C, iNOS protein expression was significantly higher in isolated compared to socially reared animals after 29 weeks of social isolation.

## Discussion

The results of the present study showed that post-weaning social isolation affected the oxidative balance, cytokine concentration, and nitric oxide production. The most important finding of this study is that these changes in the brain depend on the duration of social isolation, as the alterations in NO production were seen only after longer period of social isolation.

After 10 weeks of isolation, the body weight was not changed, but it was significantly higher after a prolonged period of isolation compared with animals reared in standard conditions. It was probably due to the higher food consumption of the isolated rats compared to the socially reared rats. Similar finding was reported by Harmer and Phillips [24]. Conversely, there is mounting evidence suggesting that weight gain and schizophrenia may be connected through dysregulation of striatal neurotransmission. Dysregulation of neuroendocrine circuits and peripheral hormones (e.g., leptin) may contribute to weight gain in people with schizophrenia. A genome-wide association study of obesity revealed a significant association between obesity-related genes and schizophrenia [25]. Stress can also induce abnormalities in food intake behavior and fat storage, causing subsequent weight changes [26].

After 10 weeks of social isolation, many of the parameters in the open field test did not change in the IR groups. However, after prolonged social isolation, we observed a significantly lower number of entries to the central zone in the IR rats than in the rats. It may be result of anxious behavior in this group. Hermes *et al.* observed decreased number of entries into the central zone in the open field test, as well as decreased time spent in this area, in IR rats [27]. The measurements were performed after seven weeks of social isolation rearing. On the other hand, another study showed opposite results. Heidbreder *et al.* used male Wistar Kyoto rats reared in social isolation for 12 weeks [28]. Although isolated rats were more active during the open field test and exhibited less anxious behavior, they spent significantly less time in the central area [29].

Measurements of habituation of the acoustic startle response (A) did not show any changes after 10 weeks of isolation rearing. However, we observed significantly lower habituation of the A in IR rats compared to SR rats after prolonged social isolation. It has been shown that social isolation induced several behavioral abnormalities and deficits in PPI [1,30]. Our results from the sensorimotor gating assessment showed a tendency toward a decline PPI after 29 weeks of isolation rearing, but the difference did not reach statistical significance. Furthermore, as we described previously, decreased PPI was observed in Sprague-Dawley rats reared in social isolation [31,32].

Several studies have shown that chronic psychosocial stress may lead to the overproduction of free radicals, which caused biochemical and molecular changes in the brain and resulted in neuronal functional impairment or structural damage. Free radicals trigger the lipid peroxidation of cell membranes, which leads to impaired membrane fluidity and receptor function [33]. In present study, the concentration of conjugated dienes was determined as a marker of membrane oxidative damage and lipid peroxidation. Our results confirmed an increased concentration of CD in the brain cortex after both 10 and 29 weeks of isolation rearing, compared to the groups. Similarly, increased lipid peroxidation, increased SOD activity and decreased concentration of reduced glutathione were determined in the model of post-weaning social isolation [34]. Additionally, increased production of free radicals may activate the antioxidant system. In our experiment, protein expression of superoxide dismutase 1 was increased after 29 weeks of IR in the hippocampus. A previous study also reported elevated levels of superoxide dismutase in patients with chronic schizophrenia compared to control values [35]. Wu *et al.* demonstrated that chronic and first-episode patients had significantly higher SOD levels than normal controls. Furthermore, chronic patients exhibited higher SOD activity than first-episode patients [36].

Pathological processes in the central nervous system (CNS) are often the result of altered immune responses. In the present study, 29-week post-weaning social isolation induced increase in concentration of plasma TNFα. An increased concentration of cytokines (IL-6, IL-12, TNFα, and INF-γ) was observed in patients with schizophrenia. The enhanced cytokines concentration stimulates the activation of NF-κB signaling pathways, which is associated with neurite growth and morphology [37]. In the first phase of schizophrenia, prodromal schizophrenia, increased levels of plasmatic cytokines were observed in patients, as well as higher activation of NFκB in peripheral mononuclear cells [38].

NO has been implicated in a great number of physiological functions in the CNS, but altered NO signaling likely contributes to various pathologies [12,13]. The cerebellum is the neural structure with the highest levels of nitric oxide. The involvement of cerebellar NO/nNOS in schizophrenia appears more evident, post-mortem investigations have reported a reduction in nitrergic neurons, nNOS-containing neurons, and NOS activity in the brain of schizophrenic patients [14]. In our experiment, total NOS activity was decreased in the cerebellum of isolation-reared rats compared to socially-reared rats, only after 29 weeks. Cerebellar protein expression of nNOS was also reduced after 29-weeks of isolation. Similarly, decreased nNOS mRNA expression and severe Purkinje cell loss were observed in the cerebellum of calcium channel mutant mice, which is related to schizophrenia [39]. Yu *et al.* reported that nNOS was significantly decreased in the cerebellum of aged rats [40]. Furthermore, several studies have reported reduced circulating concentrations of the NO metabolites nitrite and nitrate in patients with schizophrenia compared to healthy controls [13]. Several mechanisms may underlie the observed reductions in cerebellar nNOS expression and NOS activity following prolonged social isolation. Chronic isolation rearing is a potent psychological stressor that activates the hypothalamic-pituitary-adrenal axis, leading to elevated glucocorticoid levels. Excess glucocorticoids have been shown to suppress nNOS expression, impair synaptic plasticity, and reduce the availability of essential NOS cofactors, such as tetrahydrobiopterin, thereby disrupting nitric oxide synthesis [41]. Additionally, chronic stress is associated with increased oxidative stress and reactive oxygen species production, which can inhibit NOS function, promote “NOS uncoupling,” and lead to further neurotoxic effects. The degeneration of Purkinje cells, which are major sources of nNOS in the cerebellum, may also contribute directly to the reduced NOS expression and activity observed.

Finally, since nNOS is tightly linked to glutamatergic neurotransmission via NMDA receptor activation, isolation-induced alterations in glutamate signaling or NMDA receptor expression could further suppress nNOS activation. It has been shown that nNOS deficiency resulted in cognitive and behavioral disturbances, characteristic for schizophrenia. A shorter isolation period did not lead to significant changes in NOS activity or nNOS protein expression in the cerebellum. Moreover, we did not find any effect of isolation rearing on iNOS protein expression in the cerebellum following 29 weeks of isolation.

Experimental models of schizophrenia have shown the upregulation of iNOS, an isoform activated in conditions of inflammation and oxidative stress, in the brain [42]. Duration specific conditions in our study revealed increased iNOS and nNOS expression in the hippocampus in 29-week groups compared with controls. Our previous study also demonstrated increased iNOS protein levels in the hippocampus following post-weaning social isolation [32]. Similarly, Zlatkovic *et al.* showed increased iNOS expression in the hippocampus and the prefrontal cortex of male rats exposed to the chronic stress of isolation [43]. It has been reported that stress is capable of lowering GSH levels associated with iNOS induction by products of oxidative stress.

## Conclusion

Our results indicate that post-weaning isolation rearing leads to duration-dependent anxiety-like behavior, impaired startle habituation, alterations in NO signaling, and oxidative/inflammatory changes. Reduced NO bioavailability in the cerebellum may reflect increased oxidative stress and inflammation, however, causal links require further analyses.

## References

[1] Fone KC, Porkess MV (2008). Behavioral and neurochemical effects of post-weaning social isolation in rodents-relevance to developmental neuropsychiatric disorders. Neurosci Biobehav Rev.

[2] Powell SB, Swerdlow NR (2023). The relevance of animal models of social isolation and social motivation for understanding schizophrenia: review and future directions. Schizophr Bull.

[3] Braff DL, Geyer MA (1990). Sensorimotor gating and schizophrenia. Human and animal model studies. Arch Gen Psychiatry.

[4] Braff DL (2010). Prepulse inhibition of the startle reflex: a window on the brain in schizophrenia. Curr Top Behav Neurosci.

[5] Rezin GT, Amboni G, Zugno AI, Quevedo J, Streck EL (2009). Mitochondrial dysfunction and psychiatric disorders. Neurochem Res.

[6] Li XR, Xiu MH, Guan XN (2021). Altered antioxidant defenses in drug-naive first episode patients with schizophrenia are associated with poor treatment response to risperidone: 12-week results from a prospective longitudinal study. Neurotherapeutics.

[7] Herken H, Uz E, Ozyurt H, Söğüt S, Virit O, Akyol O (2001). Evidence that the activities of erythrocyte free radical scavenging enzymes and the products of lipid peroxidation are increased in different forms of schizophrenia. Mol Psychiatry.

[8] Zhang XY, Tan YL, Cao LY (2006). Antioxidant enzymes and lipid peroxidation in different forms of schizophrenia treated with typical and atypical antipsychotics. Schizophr Res.

[9] Raffa M, Atig F, Mhalla A, Kerkeni A, Mechri A (2011). Decreased glutathione levels and impaired antioxidant enzyme activities in drug-naive first-episode schizophrenic patients. BMC Psychiatry.

[10] Xin L, Mekle R, Fournier M (2016). Genetic polymorphism associated prefrontal glutathione and its coupling with brain glutamate and peripheral redox status in early psychosis. Schizophr Bull.

[11] Puzserova A, Bernatova I (2016). Blood pressure regulation in stress: focus on nitric oxide-dependent mechanisms. Physiol Res.

[12] Garthwaite J (2019). NO as a multimodal transmitter in the brain: discovery and current status. Br J Pharmacol.

[13] Akyol O, Zoroglu SS, Armutcu F, Sahin S, Gurel A (2004). Nitric oxide as a physiopathological factor in neuropsychiatric disorders. In Vivo.

[14] Zinellu A, Tommasi S, Carru C, Sotgia S, Mangoni AA (2024). A systematic review and meta-analysis of nitric oxide-associated arginine metabolites in schizophrenia. Transl Psychiatry.

[15] Fatemi SH, Folsom TD, Rooney RJ, Thuras PD (2013). Expression of GABAA α2-, β1- and ε-receptors are altered significantly in the lateral cerebellum of subjects with schizophrenia, major depression and bipolar disorder. Transl Psychiatry.

[16] Faris P, Pischedda D, Palesi F, D'Angelo E (2024). New clues for the role of cerebellum in schizophrenia and the associated cognitive impairment. Front Cell Neurosci.

[17] Bottmer C, Bachmann S, Pantel J (2005). Reduced cerebellar volume and neurological soft signs in first-episode schizophrenia. Psychiatry Res.

[18] Andreasen NC, Pierson R (2008). The role of the cerebellum in schizophrenia. Biol Psychiatry.

[19] Sasabayashi D, Yoshimura R, Takahashi T (2021). Reduced hippocampal subfield volume in schizophrenia and clinical high-risk state for psychosis. Front Psychiatry.

[20] Pierce RC, Kalivas PW (2007). Locomotor behavior. Curr Protoc Neurosci.

[21] Geyer MA, Swerdlow NR (2001). Measurement of startle response, prepulse inhibition, and habituation. Curr Protoc Neurosci.

[22] Bredt DS, Snyder SH (1989). Nitric oxide mediates glutamate-linked enhancement of cGMP levels in the cerebellum. Proc Natl Acad Sci U S A.

[23] Pechánová O, Bernátová I, Pelouch V, Simko F (1997). Protein remodelling of the heart in NO-deficient hypertension: the effect of captopril. J Mol Cell Cardiol.

[24] Harmer CJ, Phillips GD (1998). Isolation rearing enhances the rate of acquisition of a discriminative approach task but does not affect the efficacy of a conditioned reward. Physiol Behav.

[25] Grimm O, Kaiser S, Plichta MM, Tobler PN (2017). Altered reward anticipation: Potential explanation for weight gain in schizophrenia?. Neurosci Biobehav Rev.

[26] Tomiyama AJ (2019). Stress and Obesity. Annu Rev Psychol.

[27] Hermes G, Li N, Duman C, Duman R (2011). Post-weaning chronic social isolation produces profound behavioral dyegulation with decreases in prefrontal cortex synaptic-associated protein expression in female rats. Physiol Behav.

[28] Heidbreder CA, Weiss IC, Domeney AM (2000). Behavioral, neurochemical and endocrinological characterization of the early social isolation syndrome. Neuroscience.

[29] Yorgason JT, España RA, Konstantopoulos JK, Weiner JL, Jones (2013). Enduring increases in anxiety-like behavior and rapid nucleus accumbens dopamine signaling in socially isolated rats. Eur J Neurosci.

[30] Murinova J, Riecansky I (2016). Neurodevelopmental rat models of schizophrenia. Act Nerv Super Rediv.

[31] Chmelova M, Balagova L, Marko M (2019). Behavioral alterations induced by post-weaning isolation rearing of rats are accompanied by reduced VGF/BDNF/TrkB signaling in the hippocampus. Neurochem Int.

[32] Vrankova S, Galandakova Z, Benko J, Cebova M, Riecansky I, Pechanova O (2021). Duration of social isolation affects production of nitric oxide in the rat brain. Int J Mol Sci.

[33] Munhoz CD, García-Bueno B, Madrigal JL, Lepsch LB, Scavone C, Leza JC (2008). Stress-induced neuroinflammation: mechanisms and new pharmacological targets. Braz J Med Biol Res.

[34] Möller M, Du Preez JL, Viljoen FP, Berk M, Harvey BH (2013). N-Acetyl cysteine reverses social isolation rearing induced changes in cortico-striatal monoamines in rats. Metab Brain Dis.

[35] Zhang XY, Zhou DF, Cao LY, Zhang PY, Wu GY (2003). Elevated blood superoxide dismutase in neuroleptic-free schizophrenia: association with positive symptoms. Psychiatry Res.

[36] Wu Z, Zhang XY, Wang H (2012). Elevated plasma superoxide dismutase in first-episode and drug naive patients with schizophrenia: inverse association with positive symptoms. Prog Neuropsychopharmacol Biol Psychiatry.

[37] Gutierrez H, Davies AM (2011). Regulation of neural process growth, elaboration and structural plasticity by NF-κB. Trends Neurosci.

[38] Dawidowski B, Górniak A, Podwalski P, Lebiecka Z, Misiak B, Samochowiec J (2021). The Role of Cytokines in the Pathogenesis of Schizophrenia. J Clin Med.

[39] Rhyu IJ, Nahm S, Hwang SJ, Kim H, Suh YS, Oda SI, Frank TC, Abbott LC (2003). Altered neuronal NOS expression in the cerebellum of calcium channel mutant mice. Brain Res.

[40] Yu W, Juang S, Lee J, Liu T, Cheng J (2000). Decrease of neuronal nitric oxide synthase in the cerebellum of aged rats. Neurosci Lett.

[41] Johns DG, Dorrance AM, Tramontini NL, Webb RC (2001). Glucocorticoids inhibit tetrahydrobiopterin-dependent endothelial function. Exp Biol Med (Maywood).

[42] Flores-Gómez GD, Apam-Castillejos DJ, Juárez-Díaz I, Fuentes-Medel E, Díaz A, Tendilla-Beltrán H (2023). Aripiprazole attenuates the medial prefrontal cortex morphological and biochemical alterations in rats with neonatal ventral hippocampus lesion. J Chem Neuroanat.

[43] Zlatković J, Filipović D (2013). Chronic social isolation induces NF-κB activation and upregulation of iNOS protein expression in rat prefrontal cortex. Neurochem Int.

